# Estimating the ‘PrEP Gap’: how implementation and access to PrEP differ between countries in Europe and Central Asia in 2019

**DOI:** 10.2807/1560-7917.ES.2019.24.41.1900598

**Published:** 2019-10-10

**Authors:** Rosalie Hayes, Axel J Schmidt, Anastasia Pharris, Yusef Azad, Alison E Brown, Peter Weatherburn, Ford Hickson, Valerie Delpech, Teymur Noori, the ECDC Dublin Declaration Monitoring Network

**Affiliations:** 1 National AIDS Trust, London, United Kingdom; 2 Sigma Research, London School of Hygiene and Tropical Medicine, London, United Kingdom; 3 Communicable Diseases Division, Federal Office of Public Health (FOPH), Bern, Switzerland; 4 European Centre for Disease Prevention and Control, Stockholm, Sweden; 5 Public Health England, London, United Kingdom; 6 Independent Consultant, London, United Kingdom; 7 Watipa, London, United Kingdom; 8 The members of the ECDC Dublin Declaration Monitoring group are listed at the end of the article

**Keywords:** Human Immunodeficiency Virus (HIV), Pre-exposure prophylaxis (PrEP), Men who have sex with Men (MSM), European Union, Europe and Central Asia

## Abstract

In 2019, only 14 European and Central Asian countries provided reimbursed HIV pre-exposure prophylaxis (PrEP). Using EMIS-2017 data, we present the difference between self-reported use and expressed need for PrEP in individual countries and the European Union (EU). We estimate that 500,000 men who have sex with men in the EU cannot access PrEP, although they would be very likely to use it. PrEP’s potential to eliminate HIV is currently unrealised by national healthcare systems.

The international community has committed to the Sustainable Development Goal of ending the AIDS epidemic by 2030 (SDG 3.3). Pre-exposure prophylaxis (PrEP) for HIV infection involves the use of antiretroviral drugs by people at high risk of acquiring HIV. The efficacy of PrEP is well-documented [1-3]. Research in New South Wales, Australia, indicates that population-level impact of PrEP on HIV can be achieved among men who have sex with men (MSM) with a targeted, accessible programme [4]. To achieve SDG 3.3, the Joint United Nations Programme on HIV/AIDS (UNAIDS) has recommended as one of its global targets that 3 million people access PrEP by 2020 [5].

Following publication of the PROUD [2] and Ipergay [3] studies in 2015, the European Centre for Disease Prevention and Control (ECDC) released an opinion that European Union (EU) countries should consider integrating PrEP into their existing HIV prevention programmes for those most at risk of HIV infection [6]. The World Health Organization (WHO) has made the same recommendation for countries globally [7]. Here we describe the progress made by countries in Europe and Central Asia (the 53 countries of the WHO European Region plus Kosovo* and Liechtenstein) in implementing PrEP and estimate the gap between PrEP access and expressed need [8] at country and at EU level.

## Monitoring PrEP implementation in Europe and Central Asia

The ECDC disseminates an annual online survey to nominated HIV focal points, usually national health authority representatives, in the 31 countries of the EU and European Economic Area (EEA) to monitor the implementation of the Dublin Declaration on Partnership to Fight HIV/AIDS and progress towards achieving SDG 3.3 [9]. Data for the remaining 24 countries in Europe and Central Asia are supplemented with Global AIDS Monitoring (GAM) indicators collected by UNAIDS. Since 2016, the survey has included questions on PrEP availability, implementation and barriers to implementation.

The most recent survey was conducted between January and March 2019. Countries were asked to respond in relation to the most recent reporting year. Of the 55 countries surveyed by ECDC and UNAIDS, 53 provided data – the exceptions being San Marino and Turkmenistan. Data reported online were validated by the countries and updated accordingly.

We also examined data on self-reported PrEP use and expressed need for PrEP from the European MSM Internet Survey (EMIS-2017), conducted in 33 languages with 127,000 MSM from 47 of the 55 countries in Europe and Central Asia between October 2017 and January 2018 [10,11]. The eight countries not covered by EMIS-2017 were: Armenia, Azerbaijan, Georgia, Kazakhstan, Kyrgyzstan, Tajikistan, Turkmenistan and Uzbekistan. When using EMIS figures, data from the four European microstates Andorra, Liechtenstein, Monaco and San Marino were merged with those from, respectively, Spain, Switzerland, France and Italy.

## Implementation of PrEP in Europe and Central Asia

**Figure 1:**
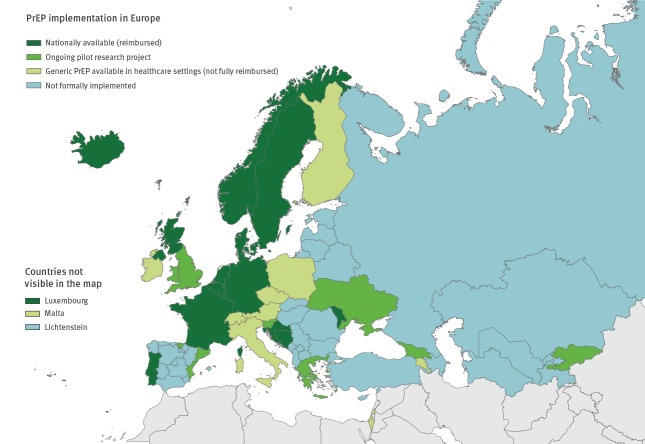
Status of PrEP implementation, Dublin Declaration monitoring in Europe and Central Asia, September 2019 (n = 53)

Data collected via Dublin Declaration monitoring provide a snapshot of a rapidly changing situation on state PrEP provision with substantial diversity across the Region (Figure 1). By 2019, 14 of 53 reporting countries reported that their national health service provided reimbursed PrEP (Belgium, Bosnia and Herzegovina, Croatia, Denmark, France, Germany, Iceland, Luxembourg, Moldova, the Netherlands, Norway, Portugal, Sweden, and Northern Ireland and Scotland within the United Kingdom (UK)), either through insurance or from the public sector. The results show that progress has been made since 2016, when only France reported that PrEP was nationally available and reimbursed [12].

PrEP: pre-exposure HIV prophylaxis.

Ten countries reported that generic PrEP was available in healthcare settings, but it was not fully reimbursed (Armenia, Austria, the Czech Republic, Finland, Ireland, Israel, Italy, Malta, Poland and Switzerland).

Six countries report PrEP availability only through pilot, research or demonstration projects at national or sub-national level (Georgia, Greece, Slovenia, Spain, Ukraine, and England and Wales within the UK). It is important to note that the degree of access to PrEP in such projects varied considerably. For example, England and Wales saw 6,000 people access PrEP in the 12 months before reporting in 2019, while Ukraine saw 125 people access PrEP in the same period.

## Barriers to implementing PrEP in Europe and Central Asia

**Figure 2:**
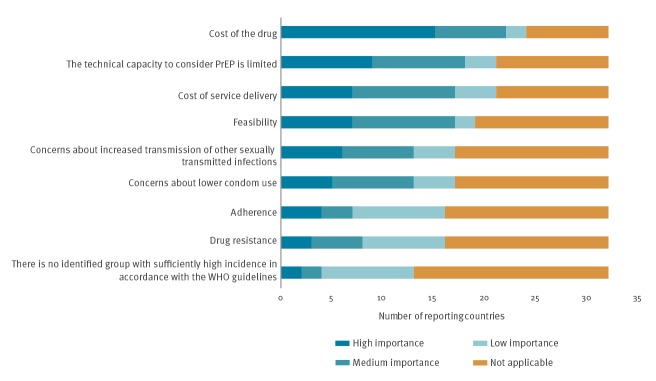
Country reported barriers to implementing PrEP, Dublin Declaration monitoring in Europe and Central Asia, 2018 (n = 32 countries)

PrEP: pre-exposure HIV prophylaxis; WHO: World Health Organization.

Of the 40 countries reporting that PrEP was not nationally available and reimbursed (including the UK because of regional differences), 32 countries provided data on barriers to implementation (Figure 2). The most commonly cited barrier to implementing PrEP was the cost of the drug. Of the 24 countries reporting high drug costs, 15 stated that cost was a high-importance barrier with a further seven citing a medium-importance barrier. Although outweighed by concerns relating to cost and service delivery, concerns about the impact of PrEP on sexual behaviours and epidemiology persisted in 18 countries.

Data in this figure are from 2018, as the question on barriers to PrEP implementation was not asked in 2019.

## Use of PrEP in Europe and Central Asia

**Figure 3:**
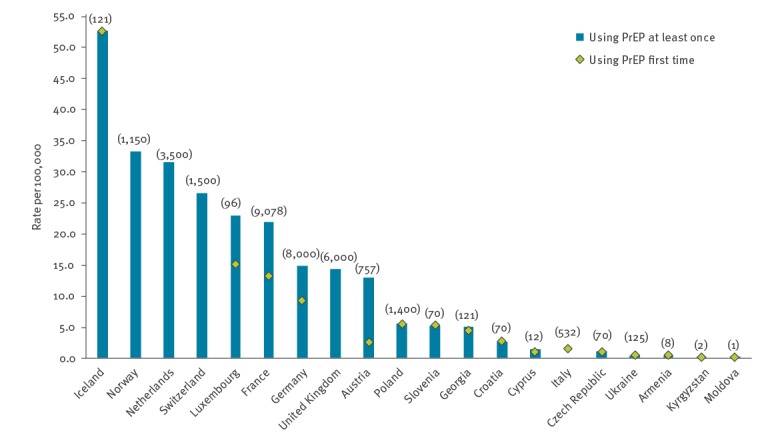
People using PrEP in the last 12 months, by rate per 100,000 adult population^a^, Dublin Declaration monitoring in Europe and Central Asia, 2019 (n = 20)

Twenty countries reported national estimates of the number of people using PrEP in the last 12 months (Figure 3). Only Switzerland and Germany reported that they were able to capture or adjust for self-sourced PrEP use within these estimates. The number and rate of people using PrEP at least once varied substantially; the number ranged from one PrEP user (Moldova) to 9,078 PrEP users (France) and the rate ranged from 0.04 to 52.5 per 100,000 adult population (aged 15–64 years). In most countries for which data were provided, the majority of PrEP users had used PrEP for the first time in the last 12 months.

PrEP: pre-exposure HIV prophylaxis.

^a^ Adult population here defined as age 15 to 64 years.

The data labels in brackets represent the numbers of people using PrEP at least once in the last 12 months. The Netherlands, Norway, Switzerland and the United Kingdom were unable to provide estimates for the number of people using PrEP for the first time in the last 12 months. Italy was unable to provide an estimate for the number of people using PrEP at least once in the last 12 months so the data label for Italy represents the number of people using PrEP for the first time in the last 12 months.

This data are correct as of March 2019.

Fifteen of the 20 countries provided data disaggregated by sex and probable transmission route, with 12 reporting that more than 90% of PrEP users were MSM. Belgium, Denmark, Ireland, Israel, Portugal, Spain and Sweden reported PrEP provision in their country but were unable to provide the number of people using PrEP.

## Estimating the PrEP gap among men who have sex with men

The EMIS-2017 collected data on both the proportion of respondents currently using PrEP and those who would be ‘very likely’ to use PrEP if they could access it. The difference between these two proportions provides an estimate of the ‘PrEP gap’ or the level of unmet need for PrEP in each country.

To calculate the PrEP gap we used EMIS-2017 data on PrEP use rather than country-reported numbers as data on use was available for a wider range of countries from EMIS-2017 and because EMIS-2017 captured PrEP use from any source whereas most country-reported data were not able to capture or adjust for self-sourced PrEP.

We chose to use the proportion of EMIS-2017 respondents who would be ‘very likely’ to use PrEP if it was available to them as the indicator of need. Studies show a positive correlation between the willingness of MSM to use PrEP and an increased risk of acquiring HIV sexually [13]. Our measure is therefore of expressed need for PrEP rather than ‘normative need’ (the criteria for PrEP access defined by experts and expressed in guidelines) [8]. As guidelines differ by country, the same group of MSM would be judged to have different levels of normative need depending on which country they lived in.

**Figure 4:**
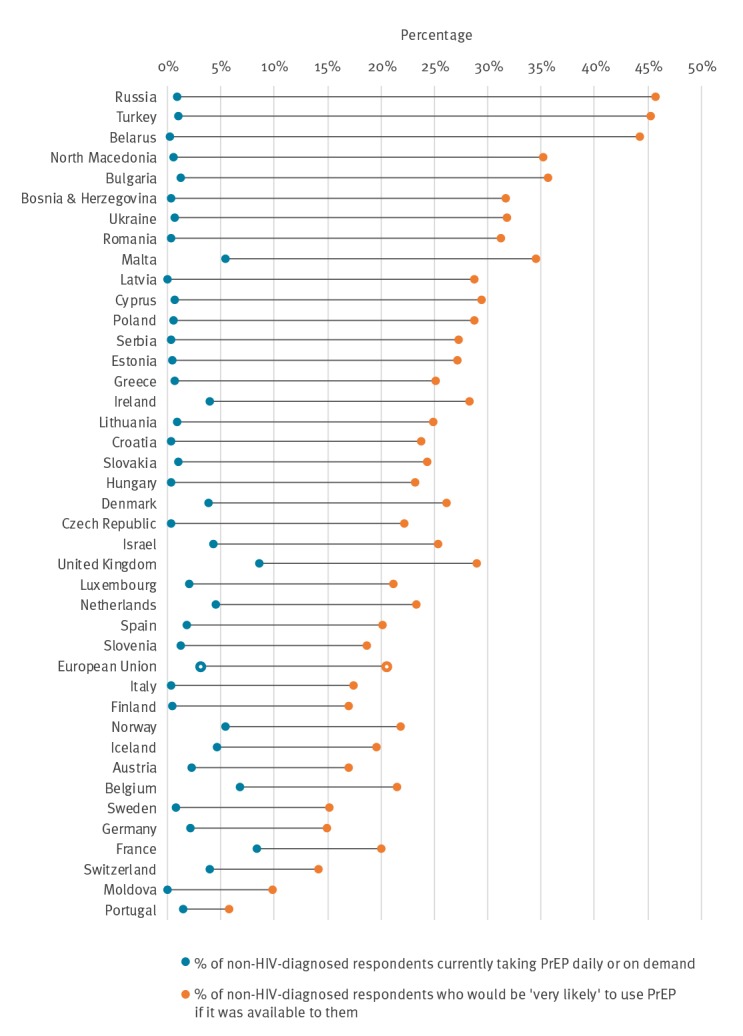
The PrEP Gap – the proportion of non-HIV-diagnosed MSM ‘very likely’ to use PrEP if accessible, compared with the proportion currently using PrEP from any source, EMIS-2017 qualifying countries, January 2018 (n=44 countries; n = 112,748 respondents)

EMIS: European MSM Internet Survey; MSM: men who have sex with men; PrEP: pre-exposure HIV prophylaxis.

The estimated PrEP gap ranged from 44.8% in Russia to 4.3% in Portugal (Figure 4). An overall estimate of the PrEP gap for the EU was calculated as 17.4%. Based on the assumption that 2.77% (95% confidence interval (CI): 2.31–3.32%) [14] of the adult male population in the EU are MSM and applying an adjustment factor of 1.6 to counter for self-selection bias [15], an estimated 500,000 (95% CI: 420,000–610,000) MSM are not currently using PrEP but would be very likely to do so if they could access it.

Countries with sample sizes < 100 respondents (Albania, Montenegro and Kosovo*) are excluded from this figure.

We assume that the fundamental reason for this gap is that in the majority of countries, easy access to free or subsidised PrEP is either not possible or not easy. Figure 4 illustrates that in the countries where formal PrEP provision occurs, the gap was smaller and in countries where there is no formal access, it was larger.

## Limitations

Country-reported data underestimate the actual number of PrEP users in some countries since many countries are not able to capture or adjust for self-sourced PrEP and because many health authorities struggle to systematically capture data on state-provided PrEP because of the diverse institutions providing it, a lack of data exchange between institutions and anonymisation of data (preventing identification of duplication).

When calculating the rate of people using PrEP per 100,000 of the adult population (with adult defined as 15–64 years) we were unable to exclude those aged 65 or over as data were not disaggregated by age.

The figure of 500,000 MSM in the EU likely to use PrEP if it were accessible may be an overestimate since EMIS-2017 used an MSM convenience sample which is known to over-represent the more sexually active. However, given that the calculation included only respondents who said they would be ‘very likely’ to use PrEP (as opposed to also including those who were ‘quite likely’) and because we used a factor to adjust for oversampling MSM at higher risk, we believe this is the best available estimate. As intention to use PrEP seemed particularly high in countries with larger populations such as Russia and Turkey and because EMIS-2017 lacks data from Central Asian countries, we restricted the calculation of the absolute numbers to the EU region.

We were able to estimate the PrEP gap only for MSM as we do not have an equivalent data source to EMIS-2017 for other key populations in need of PrEP. Where countries were able to provide disaggregated data on PrEP users, the majority indicated that less than 10% of PrEP users were either women (including trans women) or heterosexual men. Only two countries were able to provide data on PrEP users who inject drugs or engage in sex work.

## Conclusion

This paper represents the first attempt to quantify the PrEP gap for MSM in 47 EMIS-2017 qualifying countries [11] and at the EU level. At a time when provision of PrEP is rapidly increasing, these findings contribute to our understanding of PrEP implementation and use in Europe and Central Asia. Although significant progress has been made since 2016, with 14 countries now providing and reimbursing PrEP within their national health system, PrEP implementation remains variable across the Region. In order to accelerate progress towards SDG 3.3, ending the AIDS epidemic by 2030, much wider implementation of PrEP will be required.

Sex between men remains the predominant mode of HIV transmission reported in Western and Central Europe, accounting for half of all new HIV diagnoses where the transmission route is known [16]. Despite this, ca 500,000 MSM in the EU who are very likely to use PrEP are not currently able to access it. The longer the delay in access to PrEP for these men, the more HIV infections will occur.

In order to facilitate PrEP implementation across Europe and Central Asia, minimum standards on the principles of establishing PrEP programmes, monitoring and surveillance would be beneficial. These should include guidance on identifying and estimating the size of key populations in need of PrEP which can then inform programme targets. National health authorities should focus on improving accessibility of PrEP to women and heterosexual men at high risk of HIV, as well as an expansion of PrEP availability more generally.

## Note

*This designation is without prejudice to positions on status, and is in line with United Nations Security Council Resolution 1244/99 and the International Court of Justice Opinion on the Kosovo Declaration of Independence.

## References

[1] Grant RM Lama JR Anderson PL McMahan V Liu AY Vargas L Preexposure chemoprophylaxis for HIV prevention in men who have sex with men. N Engl J Med. 2010;363(27):2587-99. 10.1056/NEJMoa1011205 21091279 PMC3079639

[2] McCormack S Dunn DT Desai M Dolling DI Gafos M Gilson R Pre-exposure prophylaxis to prevent the acquisition of HIV-1 infection (PROUD): effectiveness results from the pilot phase of a pragmatic open-label randomised trial. Lancet. 2016;387(10013):53-60. 10.1016/S0140-6736(15)00056-2 26364263 PMC4700047

[3] Molina J-M Capitant C Spire B Pialoux G Cotte L Charreau I On-demand pre-exposure prophylaxis in men at high risk for HIV-1 Infection. N Engl J Med. 2015;373(23):2237-46. 10.1056/NEJMoa1506273 26624850

[4] Grulich AE Guy R Amin J Jin F Selvey C Holden J Population-level effectiveness of rapid, targeted, high-coverage roll-out of HIV pre-exposure prophylaxis in men who have sex with men: the EPIC-NSW prospective cohort study. Lancet HIV. 2018;5(11):e629-37. 10.1016/S2352-3018(18)30215-7 30343026

[5] Joint United Nations Programme on HIV/AIDS (UNAIDS). UNAIDS Strategy 2016-2021. Geneva, UNAIDS; Switzerland; 2015.

[6] European Centre for Disease Prevention and Control (ECDC). Pre-exposure prophylaxis to prevent HIV among MSM in Europe. Stockholm: ECDC; 2015. Available from: https://www.ecdc.europa.eu/en/news-events/pre-exposure-prophylaxis-prevent-hiv-among-msm-europe

[7] World Health Organization (WHO). WHO expands recommendation on oral pre-exposure prophylaxis of HIV infection (PrEP). Copenhagen: WHO; 2015. Available from: https://www.who.int/hiv/pub/prep/policy-brief-prep-2015/en/

[8] Bradshaw J. A taxonomy of social need. In: Problems and progress in medical care. McLachlan G, editor. London: Oxford University Press; 1972.

[9] European Centre for Disease Prevention and Control (ECDC). Dublin Declaration monitoring- 2018 progress. Stockholm: ECDC; 2018. Available from: https://www.ecdc.europa.eu/en/all-topics/hiv-infection-and-aids/prevention/monitoring-implementation-dublin-2018

[10] The EMIS Network. EMIS-2017 – The European Men-Who-Have-Sex-With-Men Internet Survey. Key findings from 50 countries. Stockholm: European Centre for Disease Prevention and Control; 2019.Available from: https://www.ecdc.europa.eu/en/publications-data/emis-2017-european-men-who-have-sex-men-internet-survey

[11] Weatherburn P Hickson F Reid DS Marcus U Schmidt AJ The European Men-Who-Have-Sex-With-Men Internet Survey (EMIS-2017): design and methods. Sex Res Soc Policy. Forthcoming. 10.1007/s13178-013-0119-4

[12] European Centre for Disease Prevention and Control (ECDC). Evidence brief: Pre-exposure prophylaxis for HIV prevention in Europe. Stockholm: ECDC; 2016. Available from: https://www.ecdc.europa.eu/en/publications-data/ecdc-evidence-brief-pre-exposure-prophylaxis-hiv-prevention-europe

[13] Bourne A Alba B Garner A Spiteri G Pharris A Noori T Use of, and likelihood of using, HIV pre-exposure prophylaxis among men who have sex with men in Europe and Central Asia: findings from a 2017 large geosocial networking application survey. Sex Transm Infect. 2019;95(3):187-92. 10.1136/sextrans-2018-053705 30612107 PMC6580743

[14] Mercer CH Tanton C Prah P Erens B Sonnenberg P Clifton S Changes in sexual attitudes and lifestyles in Britain through the life course and over time: findings from the National Surveys of Sexual Attitudes and Lifestyles (Natsal). Lancet. 2013;382(9907):1781-94. 10.1016/S0140-6736(13)62035-8 24286784 PMC3899021

[15] Marcus U Hickson F Weatherburn P Schmidt AJ EMIS Network Estimating the size of the MSM populations for 38 European countries by calculating the survey-surveillance discrepancies (SSD) between self-reported new HIV diagnoses from the European MSM internet survey (EMIS) and surveillance-reported HIV diagnoses among MSM in 2009. BMC Public Health. 2013;13(1):919. 10.1186/1471-2458-13-919 24088198 PMC3850943

[16] European Centre for Disease Prevention and Control and World Health Organization Regional Office for Europe (WHO/Europe). HIV/AIDS surveillance in Europe 2018 – 2017 data. Copenhagen: WHO/Europe; 2018. Available from: https://www.ecdc.europa.eu/en/publications-data/hivaids-surveillance-europe-2018-2017-data

