# Erratum for Euro Surveill. 2019;24(41)

**DOI:** 10.2807/1560-7917.ES.2019.24.46.1911141

**Published:** 2019-11-14

**Authors:** Eurosurveillance editorial team

**Affiliations:** 1 European Centre for Disease Prevention and Control (ECDC), Stockholm, Sweden

In the article ‘Estimating the ‘PrEP Gap’: how implementation and access to PrEP differ between countries in Europe and Central Asia in 2019’ by Hayes et al., published on 10 October 2019, the members of the ECDC Dublin Declaration Monitoring group were not listed in the published version. The list of names was added on 14 November 2019.

